# Tuning the Solubility Parameters of Carbon Nanotubes by Means of Their Adducts with *Janus* Pyrrole Compounds

**DOI:** 10.3390/nano10061176

**Published:** 2020-06-16

**Authors:** Daniele Locatelli, Vincenzina Barbera, Luigi Brambilla, Chiara Castiglioni, Annalisa Sironi, Maurizio Galimberti

**Affiliations:** 1 Department of Chemistry, Materials and Chemical Engineering “G. Natta”, Politecnico di Milano, Via Mancinelli 7, 20131 Milano, Italy; daniele.locatelli@polimi.it (D.L.); luigi.brambilla@polimi.it (L.B.); chiara.castiglioni@polimi.it (C.C.); 2 Eptatech SRL, Via De Gasperi 1, 22070 Luisago (CO), Italy; annalisa.sironi@eptatech.com

**Keywords:** carbon nanotubes, functionalization, solubility parameters, pyrrole compounds, Paal–Knorr reaction

## Abstract

The solubility parameters of multiwalled carbon nanotubes (CNTs) was tuned via their chemical modification with pyrrole compounds (PyCs), by means of a simple and sustainable methodology. PyCs were synthesized with high atom efficiency through the Paal–Knorr reaction of primary amines with 2,5-hexanedione, in the absence of solvents and catalysts. Methylamine, 1-dodecylamine, 2-amino-1,3-propanediol, and 3-(triethoxysilyl)propan-1-amine were selected. PyCs are characterized by two moieties, the pyrrole ring and the substituent of the nitrogen atom, and can be considered as *Janus* molecules. The functionalization of CNTs occurred with a high yield by simply heating CNTs and PyC. The whole reaction pathway did not produce any waste and was characterized by a carbon efficiency up to almost 100%. Thanks to the variety of PyC chemical structures, the CNT solubility parameter was modified in a pretty broad range of values, in the expected direction. Stable CNT dispersions were prepared in different solvents. From the aqueous dispersion, coating layers were prepared with high electrical conductivity, larger with respect to a top commercial product. The “pyrrole methodology” reported here is based on one reaction and allows almost infinite variations of the CNT solubility parameter, thus promoting their compatibility with target matrices and allowing the preparation of nanocomposite materials with improved properties. This work thus paves the way for a highly efficient exploitation of CNTs.

## 1. Introduction

sp^2^ carbon allotropes are attractive materials. Today, carbon black [1] is still one of the ten most important chemical products and the discovery of fullerenes [2] “stimulated the creativity and imagination of scientists and paved the way to a whole new chemistry and physics of nanocarbons” [3]. In the past few decades, new families of sp^2^ carbon allotropes have become available: carbon nanotubes (CNTs), both single [4,5] and multi-walled [6,7], graphene [8,9,10,11] or graphitic nanofillers made by few layers of graphene [12,13,14,15]. As has been written, “Many exciting challenges remain. There are various new allotropes to be synthesized, and there are major challenges in combining basic low-dimensional forms into more complex 3D architectures” [16].

CNTs have a unique combination of electrical, thermal and mechanical properties. For example, it has been shown, both experimentally and theoretically, that single walled CNTs have a very high tensile modulus (estimated in the range from 1 to 5 TPa), tensile strength (in the range from 50 to 200 GPa), high elasticity, and resilience [17,18,19,20]. CNTs find applications in the fields of superconductors [21], electrochemical capacitors [22], electromechanical actuators [23], photovoltaic devices [24,25], nanowires [26], and in nanocomposite materials [27,28,29].

CNTs are usually available as highly entangled bundles and their dispersion and alignment in a matrix is indeed very difficult. Moreover, the compatibilization of CNTs with the matrix depends on the solubility parameters of both tubes and matrix. An effective way to overcome these problems is the functionalization of CNTs and several methods have been successfully developed. Functionalization can be classified as covalent [30,31,32,33,34,35,36,37] and non-covalent [30,34,35,36]. The ideal functionalization method should allow the tuning of the solubility parameter of CNTs, promoting their disentanglement and dispersion in a large variety of matrices. Functional groups should be firmly bound to CNTs, without altering the structure of the carbon substrate and leaving as much surface as possible free and available for further interactions. 

In our group, the research was inspired by these objectives, aiming at developing a sustainable functionalization method, in line with the basic principles of green chemistry [38], based on easily available chemicals, ideally biosourced [39,40] and economically feasible. Moreover, the main goal was to identify a method able to promote the functionalization of most, if not all, the families of sp^2^ carbon allotropes. Functionalization of graphene layers, carbon black and CNTs was achieved with pyrrole compounds (PyCs) obtained from the Paal–Knorr reaction of a primary amine with a diketone, 2,5-hexanedione (2,5-HD) [41,42,43,44]. The synthesis of the PyCs was performed in the absence of solvents and catalysts, by simply mixing and heating the reagents, with water as the only co-product. The values of atom economy were up to: 82.5% for 2-(2,5-dimethyl-1*H*-pyrrol-1-yl)propane-1,3-diol (serinol pyrrole, SP), 75% for 1,2,5-trimethyl-1*H*-pyrrole (TMP), 88% for 1-dodecyl-2,5-dimethyl-1*H*-pyrrole (DDcP), and 89.6% for 2,5-dimethyl-1-(3-(triethoxysilyl)propyl)-1*H*-pyrrole (APTESP). The yields of reaction were: 96% for SP, 82% for TMP, 86% for DDcP and 80% for APTESP. The values of atom efficiency were instead up to: 81% for SP, 61% for TMP, 86% for DDcP, and 74% for APTESP [41,42,43]. The selected sp^2^ carbon allotrope was functionalized by simply mixing it with PyC and giving either mechanical or thermal energy [41,42,43,44]. In the latter case, it was shown [41,42,43,44] that the bulk structure of the carbon substrate remained substantially unaltered. Very high functionalization yields were reported [42], larger than 80% and, in some cases, than 95%. As the functionalization mechanism, a domino reaction has been devised [44]: a carbocatalyzed oxidation of the PyC followed by a Diels-Alder cycloaddition, with the formation of covalent bonds, between the carbon substrate and the PyC, essentially located at the edges of the former. Most available data are based on graphene layers as the carbon substrate. Preliminary indications have been reported [42] that the functionalization with different PyCs leads to the modification of the solubility parameter of graphene layers within a broad range of values.

**Figure 1:**
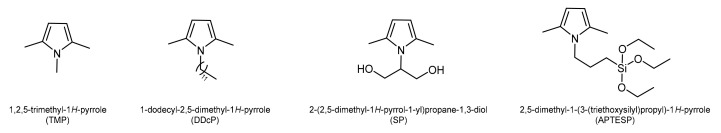
Chemical structures, IUPAC names and acronyms of pyrrole compounds (PyCs).

The objective of the present work was the functionalization of multiwalled CNTs (which will be indicated in the following as CNTs) with PyCs obtained from 2,5-HD and primary amines, such as: methylamine, 1-dodecylamine, 2-amino-1,3-propanediol, and 3-(triethoxysilyl)propan-1-amine. Chemical structures, IUPAC names, and acronyms of PyCs are in Figure 1. 

The functionalization of CNTs with PyCs was aimed at modifying their solubility parameter. As it is possible to observe in Figure 1, PyCs are characterized by two different moieties: the pyrrole ring and the substituents of the nitrogen atom. PyCs can be considered as a Janus molecule [45,46], which means a molecule with two “faces” and a dual reactivity. In ancient Rome, the god Janus was represented with two faces in one body. The two-faced Roman god inspired the definition Janus for micro- and nano-particles with at least two chemically or physically different surfaces [45,46] and also for molecules with two moieties, one hydrophilic and one hydrophobic.

Indeed, in light of the above mentioned mechanism [44], the pyrrole ring should give rise to a cycloaddition reaction with CNTs and the nitrogen substituent should lead to the modification of the CNTs solubility parameter. In order to reach this goal and to have a broad range of values, a variety of substituents was selected. In scientific literature, the solubility parameters of CNTs [47,48,49,50,51], carbon fibers [52] and graphene [53] have previously been reported. A solvent able to minimize the Gibbs free energy of mixing with graphene and CNTs was proved to be able to favor the exfoliation of graphitic aggregates [49] and the debundling of CNTs, respectively. 

The functionalization of CNTs is described and the main characteristics of the CNT-PyC adducts are discussed. The characterization of the adducts was carried out by means of thermogravimetric analysis (TGA), Raman and Fourier transform infrared spectroscopy (FT-IR), wide angle X-ray diffraction (WAXD). In order to estimate the solubility parameter of CNTs and CNT-PyC, dispersion tests of CNTs in many different solvents were performed and the qualitative results were used to estimate the Hildebrand [54] and Hansen solubility parameters (HSP) [55] and the Hansen sphere. A first example of the applications made possible by the functionalization of CNTs with PyCs is thereby presented. Coating layers were prepared with aqueous dispersions of CNTs modified with serinol pyrrole (SP) (see Figure 1) and the electrical conductivity was measured. The hypothesis was that the functionalization in a peripheral position and the unaltered bulk structure of the carbon material would lead to very good electrical properties. A comparison with a commercial dispersion of CNTs is presented.

## 2. Experimental 

### 2.1. Materials

#### 2.1.1. Multi Walled Carbon Nanotubes (CNTs)

CNTs are NANOCYL^®^ NC7000™ series from Nanocyl (Sambreville, Belgium), with carbon purity of 90%, average length of about 1.5 µm, average external diameter of 9.5 nm, and BET (Brunauer Emmett Teller measurement) surface area of 275 m^2^/g. 

#### 2.1.2. Reagents for PyCs Synthesis

2,5-hexanedione (2,5-HD), methylamine, 1-dodecylamine, 2-amino-1,3-propanediol, and 3-(triethoxysilyl)propan-1-amine were purchased from Sigma-Aldrich (St. Louis, MI, USA) and used without further purifications.

#### 2.1.3. Ingredients for the Preparation of Coating Layers

Carboxymethylcellulose sodium salt (average MW 250000, degree of substitution 0.7) was from Fluka Chemie AG (Buchs, Switzerland). Propylene glycol (Sigma-Aldrich Corp., St. Louis, MI, USA), polycarbonate emulsion (Relca PU-447, from Stahl Holdings B.V., Waalwijk, Netherlands), and Carbobyk 9870 (BYK-Chemie GmbH, Wesel, Germany) were used as received.

### 2.2. Synthesis of PyCs

The synthesis and characterization of the following PyCs: 2-(2,5-dimethyl-1*H*-pyrrol-1-yl)propane-1,3-diol (SP), 1-dodecyl-2,5-dimethyl-1*H*-pyrrole (DDcP), and 1-(3-(triethoxysilyl)propyl)-2,5-dimethyl-1*H*-pyrrole (APTESP) has been described elsewhere [45]. FT-IR spectra of synthetized PyCs are reported as Supplementary Materials in Figure S2. The synthesis of 1,2,5-trimethyl-1*H*-pyrrole (TMP) is reported as follows.

A mixture of 2,5-HD (5.51 g; 48.3 mmol) and methylamine 40% v/v in water (3.75 g; 48.3 mmol) was poured into a 250 mL round bottomed flask equipped with a magnetic bar. The mixture was then stirred at 130 °C for 4 h. After this time the reaction mixture was cooled to room temperature. 4.3 g of pure product were isolated. ^1^H-NMR (CDCl_3_, 400 MHz); *δ* (ppm) = 5.56 (s, 2H, C*H*), 3.59 (s, 3H, N-C*H*_3_), 2.14 (s, 6H, C*H*_3_). ^13^C-NMR (CDCl_3_, 100 MHz); *δ* (ppm) = 127.6; 105.6; 32.4; 12.8.

Yields of the syntheses were: SP = 95%; DDcP = 82%; TMP = 80%; APTESP = 80%. The atom efficiency were, respectively: SP = 85%; DDcP = 80%; TMP = 85%; APTESP = 90%.

### 2.3. Preparation of CNT-PyC Adducts

#### General Procedure

In a 50 mL round bottomed flask equipped with a magnetic bar, CNTs (800 mg, 11.0 mmol) and 5 mL of acetone were put in sequence. The system was sonicated for 10 min (ultrasonic bath, 260W), and then the selected PyC (15% in mass) was added into the flask. The suspension was sonicated again under the same conditions for 15 min, and then the solvent was removed under vacuum.

The CNT-PyC black powder was heated at 180°C for 3 h. After this time, the mixture was quantitatively transferred in a funnel with a sintered glass disc, washed with acetone (50 mL), and then recovered and weighed.

The functionalization yields were estimated moving from TGA data. Adducts were obtained as black powders. The functionalization yield was determined through the following equation:

(1) $$Functionalization\;Yield\;\left(\%\right)=100\times \frac{PyC\;mass\;\%\;in\;\left(CNT-PyC\;adduct\right)\;after\;acetoner\;washing}{PyC\;mass\;\%\;in\;\left(CNT-PyC\;adduct\right)\;before\;acetone\;washing}$$

### 2.4. Characterization of CNT-PyC Adducts

#### 2.4.1. Thermogravimetric Analysis (TGA)

TGA tests under N_2_ flowing (60 mL/min) were performed with a Mettler TGA SDTA/851 instrument (Mettler-Toledo S.p.A., Milano, Italy) according to the ISO9924-1 standard method. Samples (10 mg) were heated from 30 °C to 300 °C at 10 °C/min, kept at 300 °C for 10 min, and then heated up to 550°C at 20 °C/min. After being maintained at 550 °C for 15 min, they were further heated up to 900°C and kept at 900 °C for 30 min under air flowing (60 mL/min).

#### 2.4.2. Fourier Transform-Infra Red Spectroscopy (FT-IR)

FT-IR absorption spectra were recorded in transmission mode on a FT-IR Nicolet Nexus spectrometer coupled with a Thermo-Electron FT-IR Continuμm IR microscope (resolution: 4 cm^−1^; scans: 128) using a diamond anvil cell (DAC) accessory. Both instruments were provided by Thermo Fisher Scientific Inc. (Monza, Italy).

#### 2.4.3. Raman Spectroscopy

Raman spectra of powder samples were recorded with a Horiba Jobin Yvon Labram HR800 dispersive Raman spectrometer (Horiba Italia S.R.L., Roma, Italy) equipped with an Olympus BX41 microscope and a 50× objective (resolution: 2 cm^−1^; acquisition time: 30 s; 4 accumulations; Olympus Italia S.R.L., Segrate, Italy). The excitation line at 514.5 nm of an Ar^+^ laser was kept at 0.5 mW in order to prevent possible photo-induced thermal degradation of the samples. 

#### 2.4.4. Wide Angle X-ray Diffraction (WAXD)

Wide angle X-ray diffraction patterns were obtained in reflection, with an automatic Bruker D8 Advance diffractometer (Bruker Corporation, Billerica, MA, USA), with nickel filtered Cu–K_α_ radiation. Patterns were recorded in 10° ≤ 2*θ* ≤ 100°, with 2*θ* being the peak diffraction angle. 

The *D_hkℓ_* correlation length, in the direction perpendicular to the *hkℓ* crystal graphitic planes, was determined applying the Scherrer equation:

*D_hkℓ_ = Kλ*/(*β_hkℓ_* cos *θ_hkℓ_*), (2)

where *K* is the Scherrer constant, *λ* is the wavelength of the irradiating beam (1.5419 Å, Cu-K_α_), *β_hkℓ_* is the width at half height, and *θ_hkℓ_* is the diffraction angle. The instrumental broadening, *b*, was determined by obtaining a WAXD pattern of a standard silicon powder 325 mesh (99%), under the same experimental conditions. The width at half height of the peak, *β_hkℓ_* = (*B_hkℓ_* −  *b*) was corrected, for each observed reflection with *β_hkℓ_* < 1°, by subtracting the instrumental broadening of the closest silicon reflection from the experimental width at half height, *B_hkℓ_*. 

The distance between crystallographic planes was calculated from the 2*θ* value of the reflections by applying the Bragg’s law:

2d sin *θ = nλ*, (3)

where *n* is a positive integer, and *λ* is the wavelength of the incident wave.

#### 2.4.5. High-Resolution Transmission Electron Microscopy (HRTEM)

Water suspensions of CNTs and CNT-SP were prepared, at nominal concentration of 1 mg/mL, by sonicating the suspensions with a ultrasonic bath (260 W) for 10 min. HRTEM investigations on CNTs and CNT-SP adducts taken from the sonicated suspensions, were carried out with a Philips CM 200 field emission gun microscope (Philips, Eindhoven, The Netherlands) operating at an accelerating voltage of 200 kV. A few drops of the aqueous suspensions were deposited on 200 mesh lacey carbon-coated copper grid and air-dried for several hours before analysis. During acquisition of HRTEM images, the samples did not undergo structural transformation. Low beam current densities and short acquisition times were adopted. To estimate the number of walls in CNTs visible in HRTEM micrographs, the Gatan Digital Micrograph software was used (Gatan Microscopy Suite, ver. 1.8, 2015).

### 2.5. Preparation, Stability Evaluation and Characterization of Dispersions of CNT-PyC Adducts in Different Solvents

#### 2.5.1. Preparation and Stability Evaluation

Dispersions of CNT-PyC adducts (0.5 mg/mL, 10 mL) were prepared in glass tubes. Each dispersion was sonicated for 30 min using an ultrasonic bath (260 W) and was then left at rest for one week. Qualitative evaluation of the dispersions’ stability was performed, by means of visual inspection, with the help of a lamp light put at the back of the glass tubes, one hour after the end of the sonication and after one week, at rest. In this way, settling could be easily detected.

#### 2.5.2. Calculation of the Hansen Solubility Parameters (HSP) and Hansen Solubility Sphere

The calculation of the Hansen Solubility Parameters for CNTs was performed by applying the Hansen Solubility Sphere representation of miscibility. Main concepts at the basis of Hildebrand [54] and Hansen solubility parameters [55] are described in the Supplementary Materials. 

The fitting sphere program was adapted from ref. [55] and solved in MATLAB environment using the Nelder-Mead simplex algorithm. The algorithm is detailed in Figure S5 in the Supplementary Materials.

### 2.6. Preparation of CNT-Based Coating Layers from Water Dispersions, Either Based on CNT-SP or Commercially Available

CNT-SP based dispersion: 0.40 g of CNT-SP adduct were weighed in a round bottomed flask, 8 mL of distilled water, 0.20 mL of an aqueous solution of carboxymethylcellulose sodium salt (2% *w/w*) and 0.40 mL of propylene glycol were added. The mixture was magnetically stirred at room temperature for 2 h, then 0.98 mL of a polycarbonate emulsion were added, and stirring was performed at room temperature for another 2 h. 

Commercial CNT dispersion: 5.0 mL of a commercial aqueous CNT dispersion (Carbobyk 9870, BYK-Chemie GmbH, Wesel, Germany) (8% *w/w*) were poured in a round bottomed flask and 4.5 mL of distilled water, 0.11 mL of an aqueous solution of carboxymethylcellulose sodium salt (2% *w/w*), 0.23 mL of propylene glycol, and 0.55 mL of polycarbonate emulsion were added. The amount of ingredients was defined in order to have the same content of CNTs as in the CNT-SP based dispersion.

While still under stirring, a small volume of the selected dispersion was taken with a pipette and deposited on a Leneta Form 2A Opacity Chart already placed under the card holder of an automatic bar coater (K Control Coater, RK PrintCoat Instruments Ltd, Litlington, UK). Bars with thicknesses ranging from 30 μm to 120 μm were used. After selecting the suitable bar and setting the speed selector on 2, the dispersion was spread onto the substrate, obtaining the coating layer. The drying step was performed in an oven at 70 °C for 30 min. The thickness of the dried layer was measured with a magnetic feeler gauge.

## 3. Results and Discussion

### 3.1. Preparation of CNT-PyC Adducts

**Figure 2:**
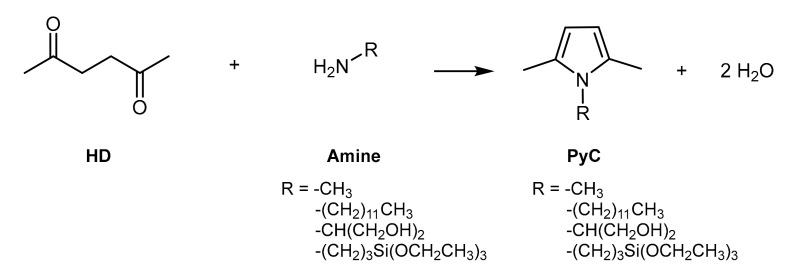
Reaction scheme for the synthesis of pyrrole-based compounds.

All the PyCs were prepared through the Paal–Knorr reaction [56,57], by reacting a primary amine with 2,5-HD, as reported in previous works [41,42] and summarized in the scheme in Figure 2.

The following primary amines were used: methylamine, n-dodecylamine, 2-amino-1,3-propanediol, 3-(triethoxysilyl)propan-1-amine. Details about synthesis can be found in the Section 2. High yields were obtained in line with previous reports [41,42]. It is worth commenting here that these syntheses, which occurred in the absence of solvents and catalysts, were characterized by a high atom economy, with water as co-product, and, thanks to the high yield, by high atom efficiency. 

**Figure 3:**
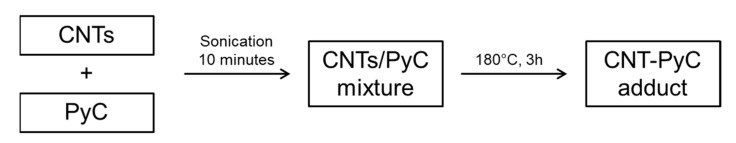
Block diagram for the preparation of carbon nanotubes (CNTs)-PyC adducts with the help of thermal energy.

In this work, functionalization reactions of CNTs with PyCs shown in Figure 2 (whose acronyms are found in Figure 1) were performed by adopting the experimental frame already used for preparing adducts of PyC with a high surface area graphite (HSAG), HSAG-PyC adducts [41,42]. Reaction details are in the Section 2, and the process is schematically shown in the block diagram in Figure 3. 

In brief, CNTs and the selected PyC were mixed in a low boiling solvent and (after solvent removal) the CNTs/PyC mixture was then heated for 3 h at 180 °C. A CNTs/PyC molar ratio (considering the moles of C6 rings as the theoretical moles of the graphitic substrate) equal to 100/15 was chosen and thermal energy was used to promote the formation of the adduct. No further optimization of the reaction conditions as a function of the PyC was performed. The solvent was used as it was the most efficient tool, at the lab scale, for obtaining an even dispersion of PyC on CNTs surface. Other techniques could be hypothesized in case of a process scale up. It is worth commenting that the synthesis of PyC could be carried out in situ, on top of the carbon material [58].

### 3.2. Characterization of CNT-PyC Adducts

#### Solvent Extraction and Yield of Functionalization

The CNT-PyC adducts were extracted in Soxhlet with acetone, until PyC was undetectable in the washing solvent. The efficiency of the functionalization reaction was investigated by performing TGA analysis on pristine CNTs and on adducts samples, before and after washing. As described in detail in the Section 2, the TGA was carried out under nitrogen up to 700 °C and under oxygen up to 900 °C. Thermograms can be found in the Supplementary Materials (Figure S1). Values of mass losses of CNTs and CNT-PyC adducts are listed in Table 1**.**

In Table 1 are also shown, for comparison, the results of TGA on SP. The mass loss below 200 °C can be attributed to water: SP is indeed a hygroscopic substance. The mass loss due to SP was found to be in the temperature range from 200 °C to 275 °C. 

In addition, in the case of CNTs and CNT-PyC adducts, mass loss at *T* < 200 °C was attributed to absorbed low molar mass substances, mainly water. The decomposition profile for all the samples, both CNTs and CNT-PyC adducts, reveals two main steps: in the temperature range from 200 °C to 900 °C mass loss can be attributed to the decomposition of alkenylic and oxygen-containing groups, while final decomposition, which occurs at *T* > 900 °C, is due to the reaction with oxygen. The amount of PyC in the adduct was estimated evaluating the mass loss in the temperature range from 200 °C to 900 °C. 

The functionalization yield was calculated through Equation (1) (see the Section 2). Values are collected in Table 2.

Functionalization yield was indeed high, at least 90%, for SP and DDcP as the PyC, and was remarkable, larger than 60% for the other PyCs. Such high functionalization yields allow to comment that the whole functionalization process is indeed sustainable, with a carbon efficiency, from the reagents to the final adducts, of almost 100%.

### 3.3. WAXD and Raman Analysis

**Figure 4:**
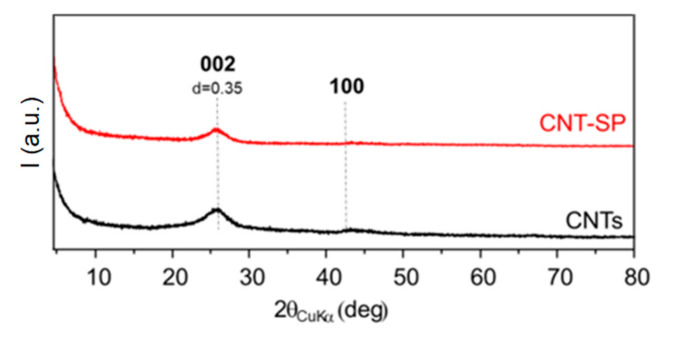
Wide angle X-ray diffraction (WAXD) patterns of pristine CNTs (black) and CNT- serinol pyrrole (SP) adduct (red).

WAXD and Raman analyses were performed with the aim to investigate the effect of the functionalization reaction on the bulk structure of CNTs. WAXD patterns and Raman spectra of CNTs and CNT-SP are provided in Figure 4 and in Figure S2, respectively. 

The WAXD patterns are shown with the 2*θ* axis value. In the scientific literature, it is usual to report the 2*θ* axis from about 10 degrees, both for graphites and chemically reduced graphite oxide [59] and for CNT [60]. In fact, the first peak is the out of plane (002) reflection, which is visible well above 20° as 2*θ* value. The rise of the line at low 2*θ* values in both CNTs and CNT-SP patterns has to be attributed to the broadening of the irradiating beam and is thus not a consequence of the functionalization reaction.

The WAXD patterns of CNTs and CNT-SP adduct are characterized by a broad (002) reflection. The graphene layers, though wrapped, are able to give rise to a crystalline stacking. The out-of-plane correlation length was estimated, by applying the Scherrer equation (see the Section 2) to the (002) reflection and was found to be about 3.0 nm for both CNTs and CNT-SP. By applying the Bragg’s equation to the 2*θ* value of the (002) peak, the distance between the wrapped graphene layers (d_002_) was calculated to be about 0.335 nm, slightly larger than those of ordered graphite samples. By dividing the out-of-plane correlation length by the interlayer distance, the number of wrapped graphene layers giving rise to a crystalline domain was estimated to be about 9, for both CNTs and CNT-SP. Hence, the stacking of wrapped layers is not altered by the functionalization reaction, which definitely does not promote the unzipping of the tubes. (100) and (110) reflections are characteristic of the order inside the graphitic planes. The (100) reflection could be detected, though hardly, in the patterns of CNTs and of CNT-SP. The absence of (101) and (112) reflections indicates the disorder in the relative position of the wrapped graphene layers and, hence, a so-called turbostratic structure [61].

Raman spectroscopy is largely used for the study of carbonaceous materials [41,42,62,63,64,65,66,67,68]. Attention is particularly focused on two peaks named D and G, located at 1350 cm^−1^ and 1590 cm^−1^, respectively, which reveal the presence of either structural defects or confinement (e.g., by edges) of the graphitic layers and the bulk crystalline graphite. Raman spectroscopy has been used in order to prove the occurring of functionalization in sp^2^ carbon materials and the reports available in the scientific literature demonstrate that the results do depend on the type of the carbon allotrope. In ref. [33], by comparing the spectra of pristine single walled CNTs (SWCNTs) and SWCNTs modified by means of 1,3-dipolar cycloaddition, it was observed that functionalization of SWCNTs brought about the increase of the intensity of the D band with respect to that of the G band. Raman spectroscopy was also applied to study the functionalization of CNTs [69]. Pristine CNTs showed a D band with intensity larger than the one of the G band [69]. This is due to the nonplanar package of the layers and marks a great difference with the Raman spectra of SWCNTs. Since this effect overlaps with that of functionalization in functionalized CNTs, a reliable evaluation of such functionalization is considered [69] to be impossible. Indeed, the Raman spectra showed that functionalization reactions did not appreciably affect the relative ratio of D and G bands [69]. In addition, the application of the four- and five-peak models for the fitting of the D band [70], used effectively also for CNTs [71], presents great problems in distinguishing highly overlapped bands with the needed accuracy so that the identification and quantification of CNTs structural modifications is considered impracticable [69]. In particular, also by considering satellite peaks, the broad nature of the spectra and the difficult assignment of the Raman features hampers the quantitative assessment of functionalization. In order to have reliable information on structural modification of CNTs from Raman spectra, deconvolution models have been developed, with particular attention to satellite peaks [69]. In the present work, Raman spectra of CNTs and CNT-SP adduct were taken. They are shown in Figure S2 and reveal the D and G bands located at the expected wavelengths, with larger intensity for the former. As expected, the occurrence of the functionalization does not modify the Raman features in line with what reported in the literature [69]. However, it seems to be possible to comment that the functionalization of CNTs with the pyrrole compound does not lead to dramatic modification of CNT structure, as it would occur, for example, in the case of tube unzipping. In light of these findings, specific research activity would have to be performed to have a reliable answer from Raman spectroscopy on the functionalization of CNTs with the pyrrole compounds.

### 3.4. HRTEM Analysis

**Figure 5:**
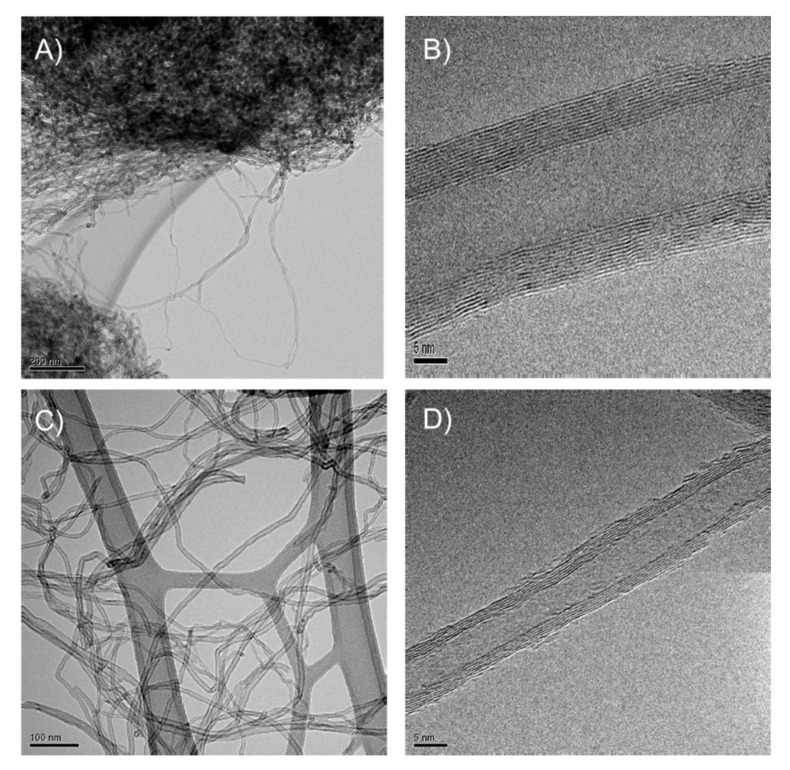
Micrographs of (**A**,**B**) CNTs and (**C**,**D**) CNT-SP adduct. Micrographs are: (**A**,**C**), low magnification bright field Transmission Electron Microscopy (TEM); (**B**,**D**) High-Resolution Transmission Electron Microscopy (HRTEM) images.

HRTEM analysis was performed on both CNTs and CNT-SP. Water dispersions were mildly sonicated with a ultrasonic bath for 10 min, samples were laid on a grid, and pictures were taken upon evaporating the solvent. Many HRTEM micrographs of each sample were taken and the representative ones are shown in Figure 5: pristine CNTs (A,B) and CNT-SP adduct (C,D).

The micrographs taken at high magnification in Figure 5B,D show a regular CNTs skeleton in both samples. The inspection of such images allowed to detect a number of wrapped CNTs layers equal to 10, a value close to the one from WAXD pattern (about 9), calculated as explained above in the text. The micrographs at low magnification reveal a different degree of aggregation for the pristine (Figure 5A) and functionalized (Figure 5C) CNTs: bundles of entangled CNTs are clearly visible in the former figure, whereas the disentanglement of CNT-SP is appreciable in the latter one. It appears that the procedure made by the functionalization and the HRTEM sample preparation steps is able to lead to CNT-PyC disentanglement. Findings shown in Figure 5 could be taken as an indication of the affinity of CNT-SP for aqueous medium.

### 3.5. FT-IR Characterization

**Figure 6:**
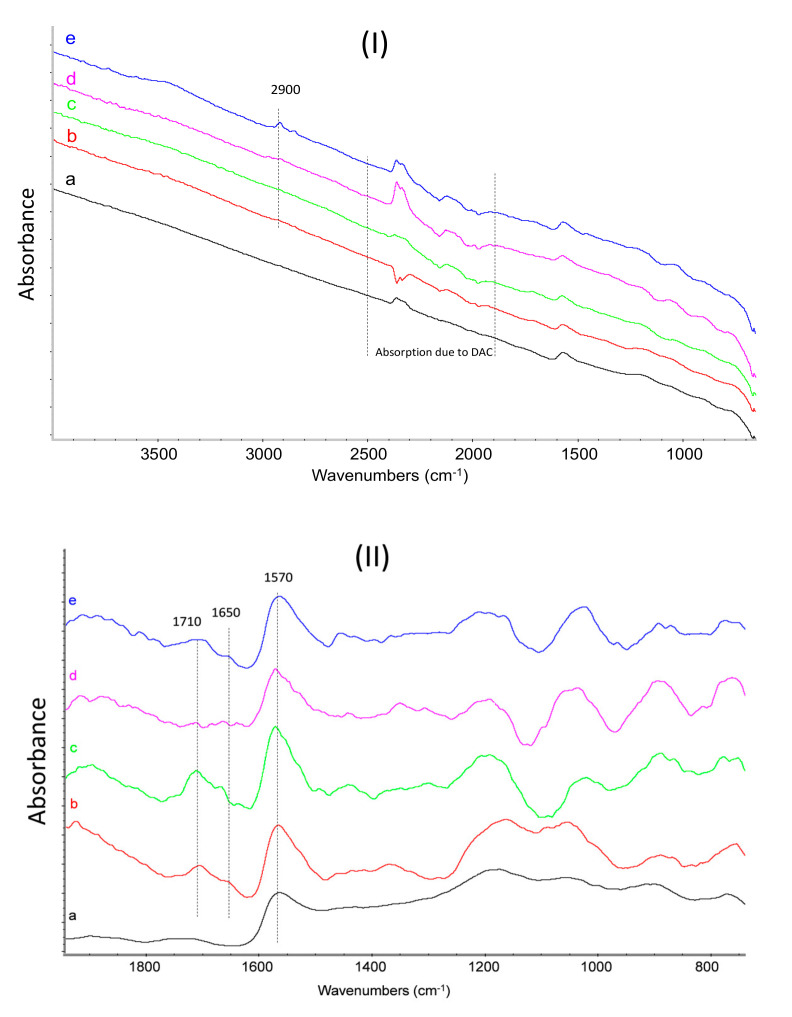
Fourier transform infrared spectroscopy (FT-IR) spectra of: (**a**) CNTs, (**b**) CNT-SP, (**c**) CNT-1,2,5-trimethyl-1*H*-pyrrole (TMP), (**d**) CNT-2,5-dimethyl-1-(3-(triethoxysisyl)propyl)-1*H*-pyrrole (APTESP), and (**e**) CNT-1-dodecyl-2,5-dimethyl-1*H*-pyrrole (DDcP). Panel (**I**): 700–3900 cm^−1^ region; Panel (**II**): fingerprint region 700–1800 cm^−1^ after baseline correction. Spectra are displayed with normalized intensity. Peaks discussed in the text and the absorption features due to the diamond anvil cell (DAC) accessory are labeled in the figure.

FT-IR spectra of the PyCs can be found in Figure S3. They reveal common features due to the vibration of the pyrrole ring and the peculiar absorption bands of the particular substituent of the nitrogen atom. The vibrational analysis of pristine CNTs and of CNT-PyC adducts after acetone extraction is presented as follows. FT-IR spectra were recorded in transmission mode in diamond anvil cell (DAC). In Figure 6, the absorption spectra are presented in the region 700–3900 cm^−1^ as recorded (panel I), and in the fingerprint region 700–1800 cm^−1^ (panel II) after baseline correction (in order to enhance the weak spectroscopic features and to ease the comparison). The chemical complexity of the samples, almost made of CNTs, leads to low and broad vibrational signals of the complexed PyCs, hampering a straightforward and detailed analysis of the spectral features. Thus, the vibrational analysis was based on the correlative recognition of the absorption bands of the main functional groups [72,73,74].

Spurious signals due to CO_2_ and diamond absorptions are identified in the region 1900–2500 cm^−1^ and are excluded from the discussion. Even if the thickness of the sample squeezed in the DAC is very low, allowing to perform a transmission experiment avoiding the presence of a dispersing agent as KBr or nujol, the presence of relatively large CNTs aggregates in the samples gives rise to a partial diffusion of the IR light. This effect produces a strong absorption background that increases toward the high wavenumber region of the spectra (see Figure 6, panel (I)). All the spectra are characterized by the strong and characteristic absorption of collective C–C stretching vibration of the CNTs, a sigmoidal shaped peak at 1570 cm^−1^. Indeed, spectra of graphitic samples recorded with DAC show this peculiar band shape, due to the interplay of diffusion/reflection of the IR light superimposed to the absorption features of graphitic particles [42,72,73,74,75]. In addition, other spectral features assigned to the organic modifier in the CNT-PyC adducts can be observed. A weak absorption assigned to C–H stretching vibrations is detectable for CNT-DDcP, CNT-SP and CNT-APTESP samples close to 2900 cm^−1^. The features ascribed to the pyrrole ring, between about 1550 cm^−1^ and 1000 cm^−1^ (see Figure 6 above and Figure S3) can be hardly detected. Two new absorption bands absent in pristine CNTs and PyCs spectra (see Figure S3) can be observed at 1710 cm^−1^ and 1650 cm^−1^ (see Figure 6, panel (II)). They can be attributed to the oxidized methyl groups in the *α* positions of the pyrrole ring, in particular to carboxylic and aldehydic groups, respectively. This aspect will be discussed in more detail in the following paragraph. 

### 3.6. On the Mechanism of the Adducts’ Formation

**Figure 7:**
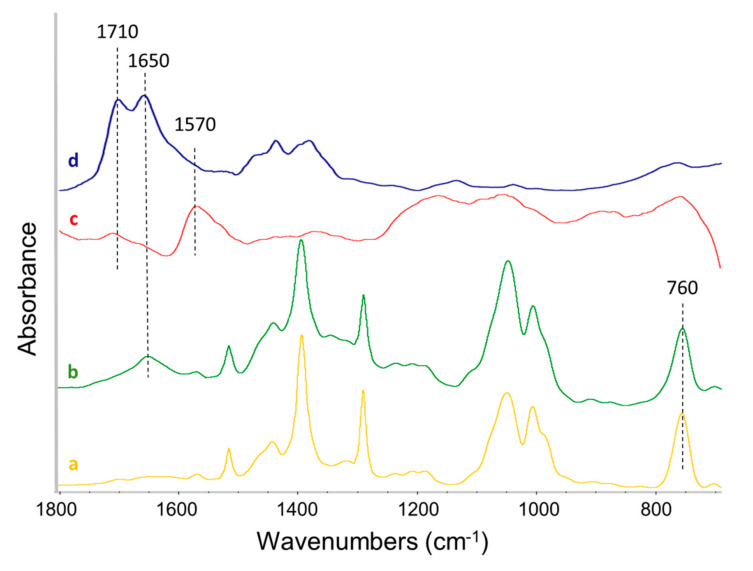
FT-IR spectra of: (**a**) SP, (**b**) SPO (1-(1,3-dihydroxypropan-2-yl)-5-methyl-1H-pyrrole-2-carbaldehyde, SP with oxidized methyl groups), (**c**) CNT-SP, and (**d**) high surface area graphite (HSAG)-TMP fingerprint region 700–1900 cm^−1^ after baseline correction. Spectra are displayed with normalized intensity.

An objective of this research was to elucidate the nature of the interaction between CNTs and PyC in the CNT-PyC adduct. To this purpose, it is useful a brief sum up of the results reported above. CNTs are functionalized with PyC, through a reaction in which the yield is up to 90% and leads to an adduct that shows mass losses at temperatures higher than those typical of a PyC. The turbostratic structure of CNTs remains substantially unaltered after the functionalization. FT-IR spectra of CNT-PyC adducts reveal the substantial absence of features due to the pyrrole moiety. On the contrary, they show the presence of two bands at 1710 cm^−1^ and 1650 cm^−1^ (less intense in CNT-APTESP spectrum), which are not typical either of PyC or of CNTs. The FT-IR spectra, in the region 1880–700 cm^−1^, of SP (a), SPO (1-(1,3-dihydroxypropan-2-yl)-5-methyl-1*H*-pyrrole-2-carbaldehyde, SP with oxidized methyl groups) (b), the CNT-SP adduct, (c) and the HSAG-TMP adduct (d) are reported in Figure 7. This latter adduct was reported and discussed in a previous publication on HSAG-PyC adducts [44] and was obtained by applying the same experimental procedure adopted in the present work for the preparation of CNT-PyC adducts. 

As already commented above, the typical features of the pyrrole ring between about 1550 cm^−1^ and 1000 cm^−1^ and the strong C–H out-of-plane bending at 760 cm^−1^ clearly visible in the spectra of SP and SPO can not to be recognized in the spectrum of the CNT-SP and HSAG-TMP adducts. These findings suggest that the aromatic pyrrole ring mostly undergoes a chemical modification as a consequence of the reaction with CNTs. In the previous work on HSAG-PyC adducts [44], very similar results were obtained and appeared to be in line with the findings from XPS: the hybridization of the nitrogen atom shifted, in a good amount, from sp^2^ to sp^3^. The two bands at 1710 cm^−1^ and 1650 cm^−1^ are visible in the spectra of both CNT-SP and HSAG-TMP. In the spectrum of SPO, a band at 1650 cm^−1^ is also visible. In the previous work on HSAG-PyC adducts [44], these bands were assigned to the oxidized methyl groups in the α position of the pyrrole ring (also by isolating and characterizing via ^1^H-NMR the oxidized pyrrole compounds). This assignment was again corroborated by XPS investigation [44], which showed the increase of the C=O/C–O ratio by increasing the reaction temperature.

The work on HSAG-PyC adducts [44] led to elaborate a mechanism for the functionalization reaction: the Domino process shown in Figure S4. The carbon substrate promotes the carbocatalyzed oxidation of the pyrrole compound and then the oxidized PyC gives rise to a cycloaddition with the graphitic material. Indeed, the disappearance or at least the weakening of the bands typical of the pyrrole ring in FT-IR spectra and the shift of hybridization of nitrogen atom, detected by XPS, were attributed to the loss of aromaticity of the heterocycle, after the cycloaddition reaction. This was considered as a key finding. It is worth adding that the reactions between HSAG and the pyrrole compounds were performed under the same experimental conditions, but in the absence of oxygen (under a nitrogen blanket). The stable adduct was not formed and the PyC was extracted with acetone [76]. The step of oxidation is indeed crucial: when an oxidized PyC and a graphitic substrate were allowed to react, the stable adduct was formed. In Figure 6 and Figure 7 above, the IR spectra have all and only the key features which should be expected on the basis of the mechanism in Figure S4. In this work, XPS investigation was not performed. However, the FT-IR findings seem to allow to hypothesize that the functionalization reactions went through the same pathway described in Figure S3. 

In scientific literature, some reactions between the modifier and the carbon substrate are supposed to occur in the light of basic chemistry rules. Mineral acids (such as HNO_3_) [77] should oxidize carbon materials and also diazonium salt and haloalkanes with zinc reagents [78,79] are expected to react with them. The point to elucidate here is if a PyC with alkyl groups in α position and a general structure shown in Figure 2, activated by means of the oxidation in benzylic position, should be expected to react with the alkenylic defects of carbon substrates. Indeed, it is widely acknowledged that oxidations typically occur on defects and tips of the carbon nanotubes [69]. 

### 3.7. Evaluation of Solubility Parameters of CNTs and CNT-PyC Adducts

To study the effect of CNTs functionalization on the compatibility of the carbon materials with matrices having different chemical nature, the Hansen solubility parameters (HSP) of CNTs and CNT-PyC adducts were estimated. The basic concepts at the basis of the Hildebrand and HSP are presented in the Supplementary Materials, with reference to the relevant scientific literature. It is worth summarizing here that the Hansen method accounts for molecular interactions between solvent and solute and leads to estimate solubility parameters based on three specific interactions: *δ_D_* (dispersion), *δ_P_* (polar), *δ_H_* (hydrogen bonding). The total *δ_T_* (Hildebrand) solubility parameter can be then calculated as the sum of the squares of the HSP. 

As anticipated in the introduction, the evaluation of solubility parameters of carbon nanomaterials has been reported in the scientific literature [47,48,49,50,51,52,53]. It is worth adding that easier percolation of CNTs in a polymer matrix and larger electrical conductivity were obtained [80] by matching the solubility parameters of CNTs and of the matrix. 

Dispersions of CNT-PyC adducts reported in Table 2 were prepared in solvents having solubility parameters *δ_T_* in the range from 14.9 (hexane) to 30 (water), belonging to the classes of alkanes, halogenated alkanes, arenes, alcohols, polar solvents. Values of HSP for these solvents can be found in Table S1. Dispersions with eight different solvents were prepared for each carbon material. In the literature, evaluation of solubility parameters of polymers is documented by using individual solvents [81,82] and the binary solvent gradient method [82]. The former approach was used in the present work. The stability of the dispersions was qualitatively studied, as described in the Section 2. In brief, dispersions were first sonicated for 30 min and then stored for one week at rest. Visual inspection of the dispersions was carried out, upon observing them one hour after the end of sonication and classifying them as “good” or “bad”: “good” was a homogenous dispersion and “bad” a mixture in which the powder settled down, completely or prevailingly. The results of the observations are shown in Table 3. Pictures of the dispersions of CNTs and CNT adducts with SP, TMP, APTESP, and DDCP, taken after one hour at rest, after the end of the sonication, are in the Supplementary Information (Figures S6–S10). Dispersions which were found homogenous after one hour at rest remained substantially stable also after one week at rest.

Evaluation of the HSP *δ_D_* (dispersion), *δ_P_* (polar), *δ_H_* (hydrogen bonding) and *δ_T_* (total) for each of the CNT-PyC adducts was made, starting from the data ofTable S1 and following the procedure described in Supplementary Materials, where the applied algorithm is described (see Figure S5). Solubility spheres, which encompass the good solvents and exclude the bad solvent, were generated. Values of the HSP and of the radius of the Hansen sphere are in Table 4. 

**Figure 8:**
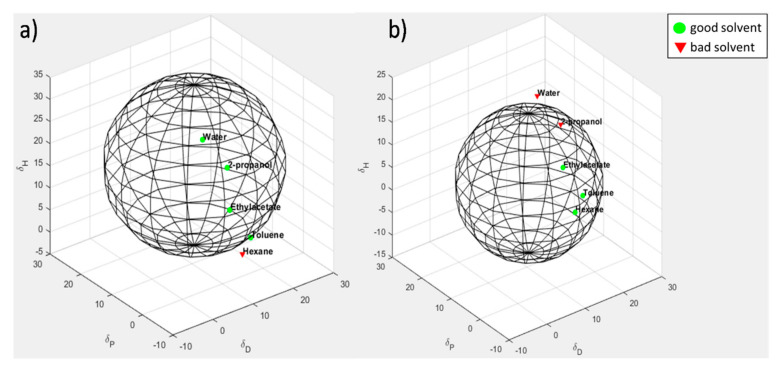
Hansen solubility sphere calculated for (**a**) CNT-SP and (**b**) CNT-DDcP. HSP (MPa1/2): for CNT-SP: *δ_D_* 12.0, *δ_P_* 11.8, *δ_H_* 15.7; for CNT-DDcP: *δ_D_* 8.5, *δ_P_* 7.5, *δ_H_* 5.3. The green circles correspond to the good solvents (within the radius of interaction); the red triangles correspond to the bad solvents (outside the sphere).

The spheres for CNT-SP and CNT-DDcP adducts are shown in Figure 8a,b, respectively. The spheres of the other adducts are reported in the Supplementary Materials (Figure S11).

On the basis of the experimental findings shown above, the following comments can be made. The functionalization of CNTs led to an appreciable increase of *δ_P_* and *δ_H_* values, for all of the PyCs, though to a different extent. To justify this finding, one could take into account that the first step of the functionalization mechanism is the oxidation of the pyrrole compound, with the formation of an aldehydic group in the α position of the pyrrole ring. Hence, an oxygenated group is present in all of the CNT-PyC adducts. The largest increase of the *δ_H_* value was observed for CNTs functionalized with serinol pyrrole: SP is the only PyC that introduces OH groups. Indeed, polar protic solvents, such as water and 2-propanol are inside the sphere for CNT-SP, in which homogeneous dispersion can thus be successfully prepared, even in ecofriendly aqueous media. It appears reasonable that, in the case of CNT-APTESP, the increase of *δ_P_* value was larger than the increase of *δ_H_* value. In the case of CNT-TMP, the largest increase of Hansen parameters was observed for *δ_P_.* It could be speculated that the carbonyl group plays an appreciable role in the case of an adduct with only a methyl group as the substituent of the nitrogen atom. In the case of CNT-DDcP, water and 2-propanol fall outside the Hansen sphere, and the largest value was obtained for *δ_D_*. In spite of the pretty limited number of experimental data, results appear in line with what could be expected in the light of the chemical structure of the PyCs and confirm that the functionalization of CNTs with different PyCs allows to tune the solubility parameter and hence the dispersibility of CNTs in various media. It is also worth emphasizing the large increase of the Hansen sphere radius (see Table 4): CNTs become compatible with a good variety of dispersing media.

### 3.8. Electrically Conductive Coating Layer with CNT-SP

**Figure 9:**
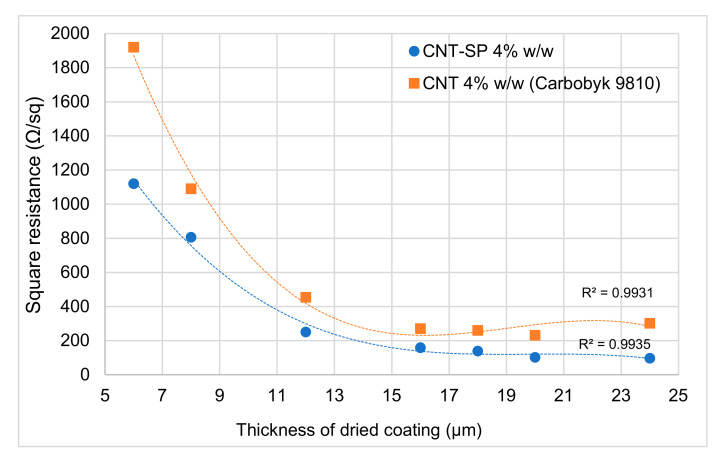
Square resistance vs. thickness of dried coating layers for the following dispersions: based on CNT-SP (blue circles), based on commercially available (Carbobyk 9810) (red squares). Points are interpolated with third grade polynomials.

There is a great interest for transparent and electrically thin conductive films and coatings, based on CNTs [83,84,85]. CNTs can replace the most used indium tin oxide (ITO) thanks to intrinsic flexibility and fatigue resistance and a large variety of applications can be envisaged. Aqueous dispersions are increasingly requested in the coating field, because of the low environmental impact. A stable dispersion of CNTs in a water-based polymeric matrix, in which the recipe is in the Section 2, was prepared, thanks to the functionalization with SP. A similar paint was obtained starting from a commercially available water-based dispersion (see Section 2, as well as Table S2 and Figures S12 and S13 in the Supplementary Materials, for its characterization), keeping the same CNT percentage weight as in the other formulation. Coating layers of different thicknesses were obtained by depositing the dispersions with a bar-coater. After drying in an oven, the homogeneity of the layers was visually ascertained and the absence of aggregates or failures verified (see pictures in Figure S14). The electric resistance of the layers was measured by means of a multimeter; then square resistance values were calculated and plotted in the graph of Figure 9, as a function of the coating layer thickness. 

Lower square resistance, at all the layers’ thicknesses, was obtained with the CNT-SP dispersion. A working hypothesis to explain this finding could make reference to the dispersibility of CNTs in water thanks to the functionalization with SP. The HRTEM micrographs at low magnifications reported above in Figure 5A,C show that the functionalization with SP improves the disentanglement of CNTs and hence the processability during the preparation of a coating layer. Indeed, the aspect of CNTs processability, as a function of the functionalization, deserves to be investigated in future experiments. Moreover, the CNT surface can be considered to remain substantially unperturbed, after the functionalization, as it was also shown by WAXD, XRD, and Raman analyses. In fact, a relatively minor amount of modifier is introduced, essentially in defective and peripheral positions. Finally, the functionalization with SP does not lead to cover the CNT surface, as in the case of the traditional surfactants. 

## 4. Conclusions

This work shows that it is indeed possible to change the solubility parameter of CNTs by means of the formation of stable adducts of CNTs with PyCs, obtained with only one type of chemical reaction, in a simple and sustainable way. PyCs were prepared with high atom efficiency through Paal–Knorr reactions carried out in the absence of solvents and catalysts. PyCs were characterized by different groups as substituent of the nitrogen atom: methyl, dodecyl, 1,3-propanediol, 3-(triethoxysilyl)propane. The functionalization reaction was performed by simply mixing and heating CNTs and the selected PyC. The whole functionalization process, from the reagents to CNT-PyC adducts is characterized by a carbon efficiency up to almost 100%. In their interaction with CNTs, PyCs acted as *Janus* molecules: the pyrrole ring gave rise to the cycloaddition reaction with CNTs and the nitrogen substituents, thanks to their different chemical nature, led to the modification of the CNT solubility parameter in a broad range of values. Estimated Hildebrand parameters were from 12.5 for CNT-DDcP to 23 for CNT-SP. It is indeed worth commenting that, in each case, the CNT solubility parameter was modified in the direction dictated by the chemical nature of PyC. Stable dispersions of CNTs were obtained in various solvents, as a function of their solubility parameter. Coating layers prepared from an aqueous dispersion of CNT-SP revealed great electrical conductivity. These results can be justified in the light of the typical features of this functionalization methodology: chemical modification occurs in peripheral positions and bulk structure of the graphitic substrate remains substantially unaltered. 

Cycloaddition reactions for the functionalization of carbon nanotubes are well known in the scientific literature [33], with plenty of examples. In most cases, they led to improve the solubility of the nanotubes. What is novel in this work is the chance of using the same reactive moiety, a pyrrole ring, for functionalizing CNTs with a large variety of functional groups. Indeed, the functional groups which can be brought on CNTs are those present in primary amines, which are commercially available and also biosourced reagents. Besides this versatility, a key novel feature of such a functionalization procedure, named as “pyrrole methodology”, is the very high atom efficiency and, in particular, carbon efficiency, with only water as the co-product. The chemical reaction here applied was shown [44] to lead to the successful modification of graphene layers through a domino process, which can be reasonably hypothesized to occur also on CNTs. In the case of HSAG, it was also shown [58] that the domino process can be triggered by mixing the primary amine and 2,5-HD on top of the carbon allotrope, providing then thermal energy. This could be definitely done also in the case of CNTs, paving the way for the scale-up of the adducts’ preparation. Since the early works on carbon nanotubes’ functionalization, it was clear the importance of promoting the solubility of the tubes, in different environments [86].

Nanocomposite materials can potentially be prepared with much improved interactions between CNTs and the matrix. The CNT functionalization technology discussed in this work paves the way for the most efficient transfer to the macroscale of the exceptional properties of CNTs. In a recent paper by some of the authors, CNTs were functionalized with the “pyrrole methodology” and were then used as nano-conveyors for cancer drug delivery [87].

## Figures and Tables

**Table 1:** Mass losses (mass %) for CNTs and CNT-PyC adducts, from thermogravimetric analysis (TGA) analyses.

Sample	Temperature Range			
	0 < *T* < 200 °C	200 < *T* < 700 °C	700 < *T* < 900 °C	*T* > 900 °C
SP	6.0	94.0 ^a^	0	0
CNTs	0.8	2.6	0.1	96.5
CNT-SP	1.8	9.6	6.5	82.0
CNT-TMP	1.6	7.5	4.9	85.9
CNT-DDcP	1.1	8.4	8.2	82.2
CNT-APTESP	1.5	7.8	3.7	87.0

^a^ Mass loss in the range from 200 °C to 275 °C.

**Table 2:** Yield of functionalization of CNTs with PyC ^a^.

Adduct	PyC in the Adduct (phc) ^b^	Functionalization Yield (%) ^c^
CNT-TMP	10.9	73
CNT-DDcP	13.3	92
CNT-SP	13.5	90
CNT-APTESP	9.8	65

^a^ CNTs/PyC= 100:15; **^b^** phc = parts of PyC per hundred parts of CNTs; ^c^ Calculated through Equation (1).

**Table 3:** Results of inspection of stability of CNTs and CNT-PyC adducts dispersions in various solvents ^a,b^.

Solvent	Samples				
	CNTs	CNT/TMP	CNT/DDcP	CNT/SP	CNT/APTESP
Alkanes: hexane heptane	GOOD n.d.	BAD n.d.	GOOD GOOD	BAD BAD	BAD BAD
Halogenated alkanes: chloroform	n.d.	BAD	BAD	n.d.	n.d.
Arenes: toluene	GOOD	GOOD	GOOD	GOOD	GOOD
Alcohols: 2-propanol 2-butanol methanol	BAD n.d. BAD	GOOD GOOD n.d.	BAD n.d. n.d.	GOOD n.d. n.d.	GOOD n.d. n.d.
Polar solvents: acetone water	BAD BAD	GOOD BAD	n.d. BAD	GOOD GOOD	GOOD BAD
Others: ethyl acetate dichloromethane	BAD BAD	GOOD n.d.	GOOD BAD	GOOD BAD	GOOD GOOD

^a^ concentration: 0.5 mg/mL; ^b^ GOOD: homogeneous dispersion was observed soon after sonication and after one week storage at rest; BAD: separation of adduct from the solvent; n.d.: not determined in that solvent

**Table 4:** Hansen solubility parameters (HSP) and sphere radius for the CNT-PyC adducts ^a^.

Sample	*δ_D_*	*δ_P_*	*δ_H_*	Radius	*δ_T_* ^b^
CNTs	17.0	1.7	1.3	3.3	17.1
CNT-TMP	8.7	12.0	5.2	15.8	15.7
CNT-DDcP	8.5	7.5	5.2	15.4	12.5
CNT-SP	11.9	11.4	15.7	18.1	22.7
CNT-APTESP	6.7	12.0	5.2	15.8	14.7

^a^ Measure unit: MPa^1/2^; ^b^ *δ_T_*^2^ = *δ_D_*^2^ + *δ*_P_^2^ + *δ_H_*^2^.

## Supplementary Materials

The following are available online at https://www.mdpi.com/2079-4991/10/6/1176/s1, TGA, Raman, FT-IR TEM and HSP details, Figure S1: Thermograms of pristine CNTs and CNT-PyC adducts, Figure S2: Raman spectra of CNTs and CNT-SP, Figure S3: FT-IR spectra of PyCs, Figure S4. Mechanism for the interaction of graphene layers with pyrrole compounds, Table S1: Hansen solubility parameters for the selected solvents, Figures S6–S10. Dispersions of the CNT-PyC adducts in different solvents, Table S2: Aqueous dispersions of CNT: based on CNT-SP and Carbobyk 9810, Figure S12. TEM and HRTEM micrographs of Carbobyk 9810 MWCNT water-based dispersion. Figure S13. WAXD pattern of Carbobyk 9810 dried dispersion, Figure S14 Coating layers of dispersions.
