# Supplementary material for: Tuning the Solubility Parameters of Carbon Nanotubes by Means of Their Adducts with Janus Pyrrole Compounds
Source: Nanomaterials (Basel). 2020 Jun 16;10(6):1176. doi: 10.3390/nano10061176 (PMC7353205; doi:10.3390/nano10061176)
Supplement: Supplementary file 1 [file nanomaterials-10-01176-s001.pdf]

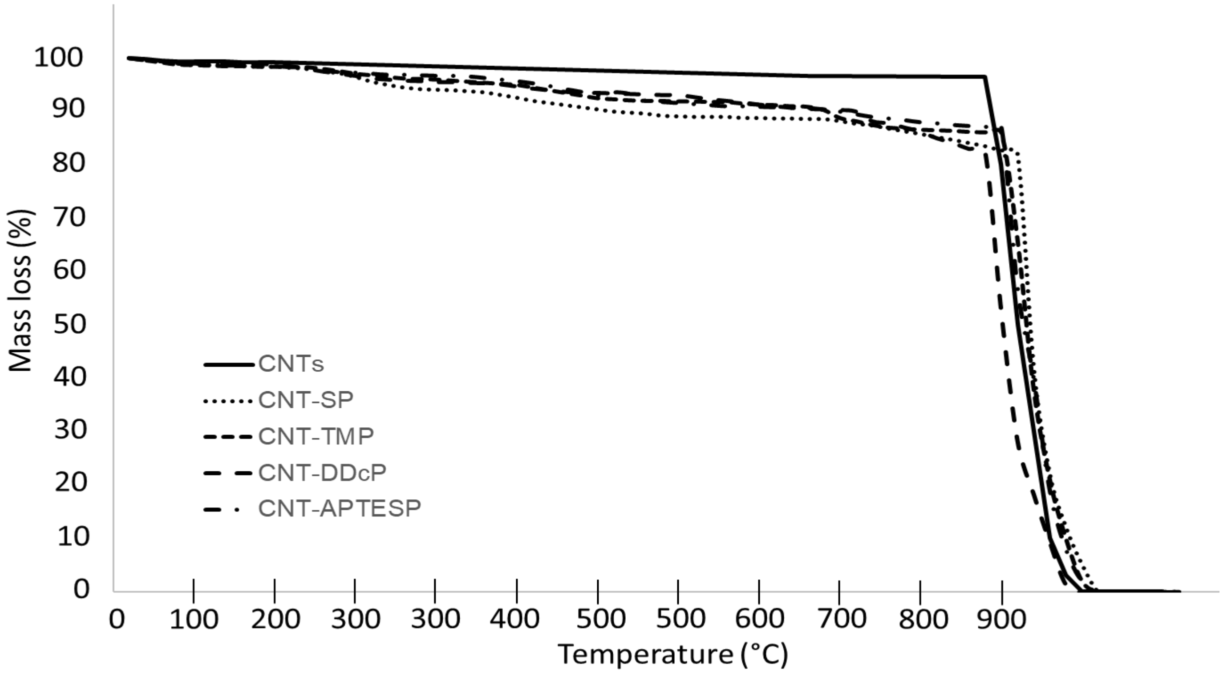

Figure S1. Thermograms of pristine CNTs and CNT-PyC adducts.

TGA analyses were performed as described in the experimental part.
Quantitative data of mass losses are in the manuscript.

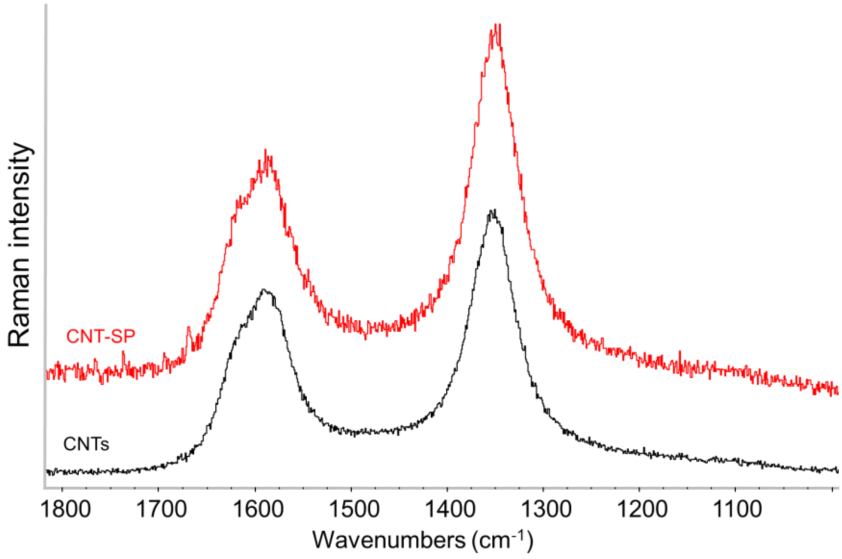

Figure S2. Raman spectra of CNTs (black) and CNT-SP (red) excited at 514 nm.

Spectra were taken as described in the experimental part.

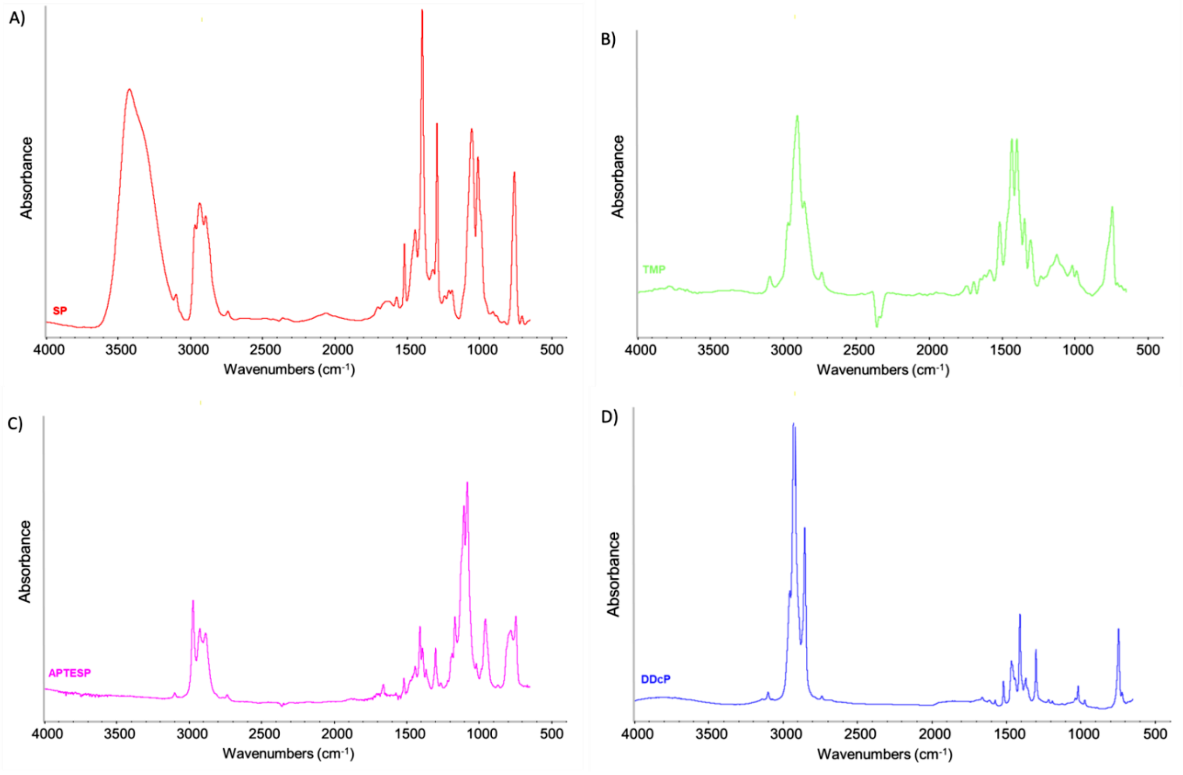

Figure S3. FT-IR spectra of: (**A**) SP, (**B**) TMP, (**C**) APTESP, and (**D**) DDCP 700–3900 cm^{-1} region after baseline correction.

PyC FT-IR spectra are reported in the 500–4000 cm^{-1} region. The typical bands of the pyrrole ring are clearly visible in the spectra of all PyCs: (1) in the range between about 1550 cm^{-1} and 1000 cm^{-1}; (2) the C–H stretching vibration of H–C=C group at 3100 cm^{-1} (strong peak); (3) out-of-plan hydrogen atom vibration at 760 cm^{-1} (weak band).

The bands at about 2900 cm^{-1} in SP spectrum (also present in TMP, APTESP, and DDcP spectra) are assigned to C–H stretching vibrations of CH_3 and CH_2 units, whereas the bands close to 1450 cm^{-1} are assigned to their bending vibration. The broad band at 3400 cm^{-1} is assigned to the stretching of hydrogen-bonded OH groups belonging to the pyrrole compound's tail. The bands around 1100–1200 cm^{-1} are attributed to the stretching vibration of C–OH functional groups.

In the spectrum of APTESP the band detected around 1200 cm^{-1} is attributed to the Si–O stretching vibrations.

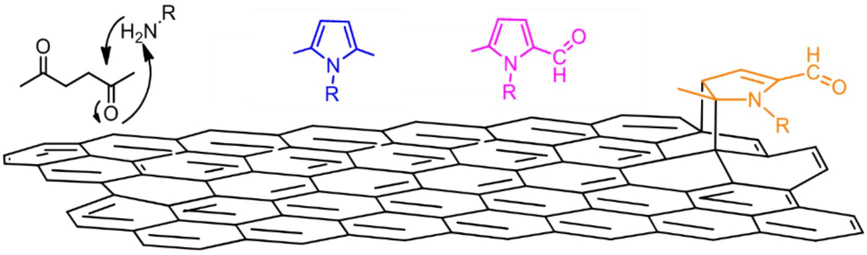

Figure S4. Mechanism for the interaction of graphene layers with pyrrole compounds.

Calculation of the Hansen Solubility Parameters and Hansen Solubility Sphere

The interaction between a solvent and a solute is governed by the following equation:

$$\Delta G_M = \Delta H_M - T\Delta S_M, \tag{S1}$$

where ΔG_M is the Gibbs free energy change of mixing, ΔH_M is the enthalpy change of mixing, T is the absolute temperature, and ΔS_M is the entropy change of mixing. A spontaneous mixing occurs when

the free energy change has a negative value, that means $\Delta G_M < 0$. When the dissolution of a solute is associated with a small positive entropy change, ΔH_M becomes the dominant factor for determining the sign of ΔG_M.

Hildebrand found that the solubility of a solute in a series of solvents was determined by the internal pressures of the solvents [Ref. S1]. Development of the Hildebrand theory was due to Scatchard, who introduced the concept of cohesive energy density [Ref. S2]. Then, Hildebrand and Scott [54] proposed to describe the enthalpy of mixing by means of the following equation:

$$\Delta H_M = V_M[(\Delta E_1^V/V_1)^{1/2} - (\Delta E_2^V/V_2)^{1/2}]^2\phi_1\phi_2, \quad \text{(S2)}$$

where V_M is the volume of the mixture; ΔE_i^V is the energy of vaporization of species i, that is the change of energy upon isothermal vaporization of a saturated liquid to an ideal gas state of infinite volume; V_i is the molar volume of species i, and ϕ_i is the volume fraction of species i in the mixture.

The cohesive energy E of a material can be defined as the difference (increase) of the internal energy per mole of a material when all of the intermolecular forces are eliminated. The cohesive energy density (CED) is the energy required to overcome all intermolecular forces per unit volume of a material and can be expressed by the following equation:

$$CED = E/V = (\Delta H_{VAP} - RT)/RT, \quad \text{(S3)}$$

where ΔH_{VAP} is the enthalpy of vaporization.

The Hildebrand solubility parameter is defined by the following equation:

$$\delta = (E/V)^{1/2} \quad \text{(S4)}$$

as the square root of the cohesive energy density.

Equation (S2) can be modified by introducing the Hildebrand solubility parameter (see Equation (S4)), obtaining the following equation:

$$\Delta H_M/V = (\delta_1 - \delta_2)^2\phi_1\phi_2, \quad \text{(S5)}$$

which gives the enthalpy of mixing per unit volume in the case of a binary mixture.

Equation (S5) shows that the enthalpy of mixing should be lower than the entropy of mixing to have spontaneous mixing. Hence, the difference in solubility parameters has to be small, to have miscibility over the whole volume fraction range.

The Hildebrand method does not consider specific interactions between molecules, such as hydrogen bonding. To overcome this limit, Hansen developed a method which accounted for molecular interactions and defined solubility parameters based on three specific interactions. In the Hansen method, the cohesive energy E is made by three contributions:

$$E = E_D + E_P + E_H. \quad \text{(S6)}$$

The contributions are due to dispersion (D), polar–polar (P), and hydrogen bonding (H) forces.

This equation can be divided by the molar volume, as follows:

$$E/V = E_D/V + E_P/V + E_H/V. \quad \text{(S7)}$$

Equation (S7) shows that the square of the total (Hildebrand) solubility parameter is the sum of the squares of the Hansen components:

$$\delta_T^2 = \delta_D^2 + \delta_P^2 + \delta_H^2. \quad \text{(S8)}$$

The solute is therefore identified by three coordinates (δ_D, δ_P and δ_H) in the Hansen parameters space, called Hansen solubility parameters (HSP). The distance between two points (e.g. a solute and its solvent) is related to their cohesive energy density difference, which is in turn related to the enthalpy of mixing. As the enthalpy of mixing is minimal for miscible substances, two points close to each other in the Hansen space correspond to miscible compounds.

To estimate the HSP of a solute i, a dispersion test is performed on different solvents j, distinguishing good solvents that provide stable solutions/dispersions from bad ones, which are not

able to give stable dispersions. Given the parameters (coordinates) of the solvents, it is possible to define a sphere, centered on the solubility parameters of the solute, which encompasses the good solvents and excludes the non-solvents. The sphere radius is defined as R_0, the radius of interaction, while the distance between the solute and the solvent is R_a, obtained from the following equation:

$$R_{a,ij}{}^2 = 4(\delta_{D,i} - \delta_{D,j})^2 + (\delta_{P,i} - \delta_{P,j})^2 + (\delta_{H,i} - \delta_{H,j})^2 \quad \text{(S9)}$$

The ratio between $R_{a,ij}$ and R_0 is defined in Equation (S10) as RED, relative energy difference. Solutes and solvents with good affinity have RED < 1.

$$RED = \frac{R_{a,ij}}{R_o} \quad \text{(S10)}$$

An optimization problem is therefore defined: the center coordinates of the Hansen solubility sphere are calculated by minimizing the radius of interaction (i.e., the distance from the coordinates of the good solvents), including the good solvents (RED < 1) and excluding the bad ones. The sphere center coordinates correspond to the three unknown HSP of the solute.

The elaboration was carried out by means of an algorithm described in the following scheme (Figure S5).

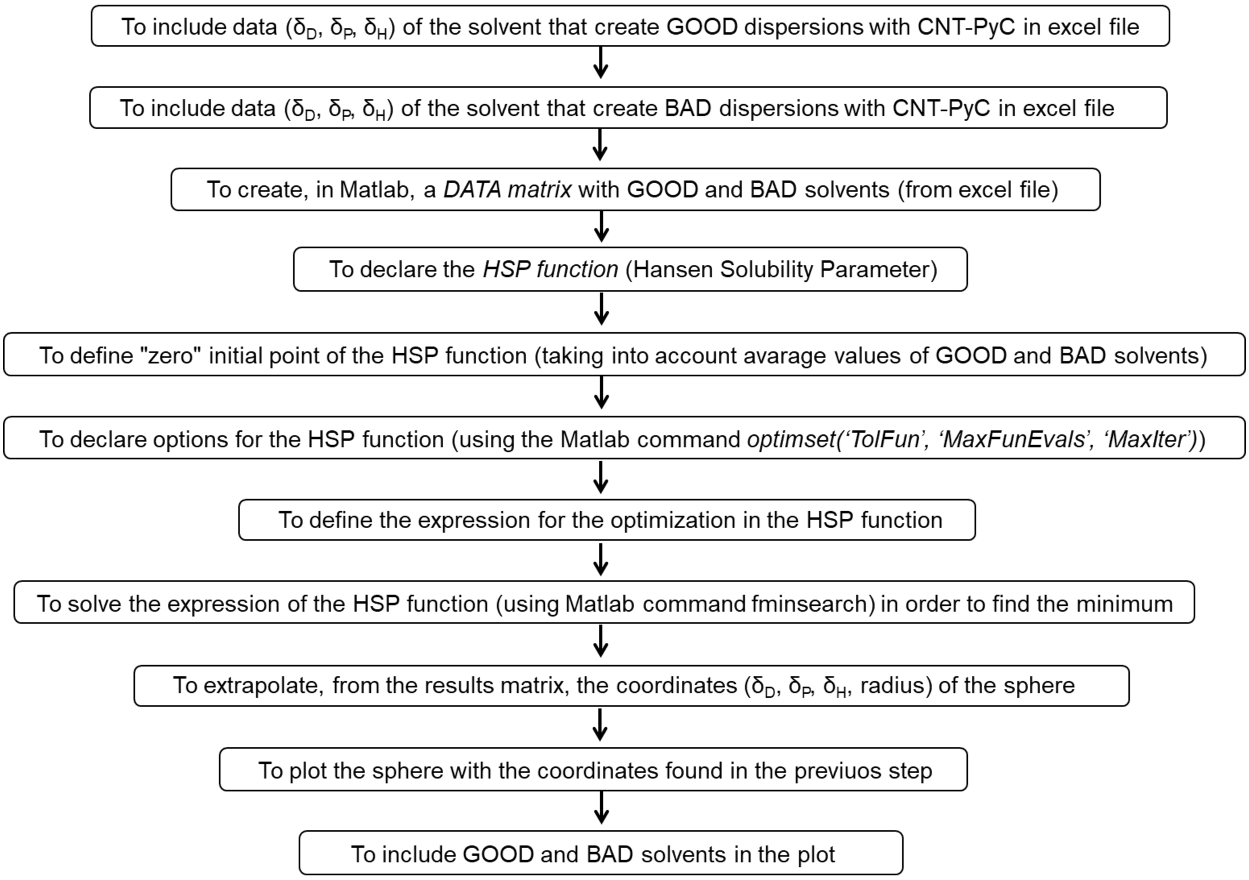

Figure S5. MATLAB algorithm used for the preparation of Hansen's sphere.

The algorithm was used in MATLAB in order to evaluate the coordinates of the spheres.

In particular, in the HSP function, a non-linear optimization through the Nelder-Mead method was done in order to obtain minimum values of the functions using the optimized coordinates of the spheres.

Table S1. Hansen solubility parameters for the selected solvents [a].

Solvent	δ_D	δ_P	δ_H	δ_T [b]
Alkanes:				
Hexane	14.9	0.0	0.0	14.9
Heptane	15.3	0.0	0.0	15.3
Halogenated alkanes:				
Chloroform	17.8	3.1	5.7	18.9
Arenes:				
Toluene	18.0	1.4	2.0	18.2
Alcohols:				
2-propanol	15.8	6.1	16.4	23.6
2-butanol	15.8	5.7	14.5	22.2
Methanol	15.1	12.3	22.3	29.6
Polar solvents:				
Acetone	15.5	10.4	7.0	19.9
Water	18.1	17.1	16.9	30.1
Others:				
Ethyl acetate	15.8	5.3	7.2	18.1
dichlorometane	18.2	6.3	6.1	20.2

[a] – Unit of measurement: $MPa^{1/2}$; [b] – $\delta_T^2 = \delta_D^2+\delta_P^2+\delta_H^2$

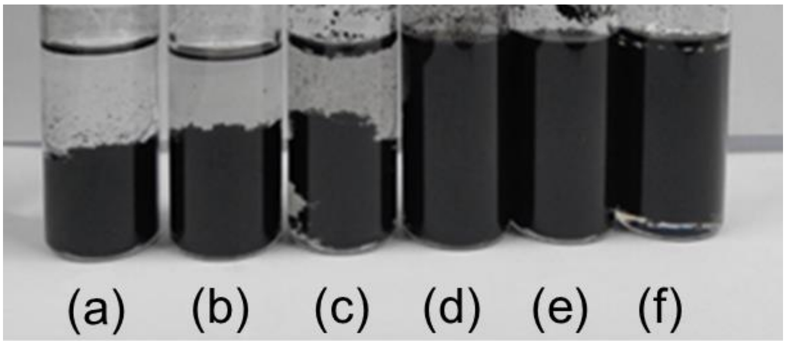

Figure S6. Dispersions of pristine CNTs in different solvents (after 1 h at rest): (**a**) water, (**b**) 2-propanol, (**c**) ethyl acetate, (**d**) toluene, (**e**) hexane, and (**f**) dichloromethane. Samples (**d**–**f**) were considered to be "GOOD"; samples (**a**,**b**,**c**) were considered to be "BAD".

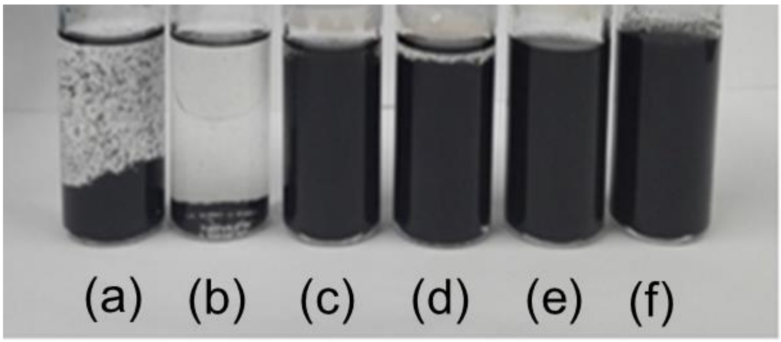

Figure S7. Dispersions of the CNT-TMP adduct in different solvents (after 1 h at rest): (**a**) water, (**b**) hexane, (**c**) 2-propanol, (**d**) ethyl acetate, (**e**) toluene, and (**f**) acetone. Samples (**c**–**e**), and (**f**) were considered to be "GOOD"; samples (**a**,**b**) were considered to be "BAD".

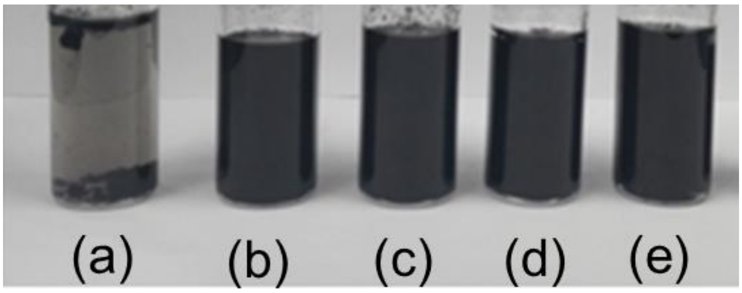

Figure S8. Dispersions of the CNT-DDcP adduct in different solvents (after 1 h at rest): (**a**) water, (**b**) ethyl acetate, (**c**) toluene, (**d**) hexane, and (**e**) 2-propanol. Samples (**b**–**d**), and (**e**) were considered to be "GOOD"; sample (**a**) was considered to be "BAD".

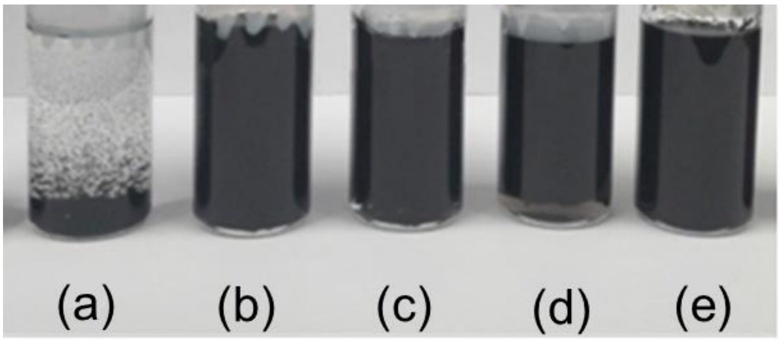

Figure S9. Dispersions of CNT-SP adduct in different solvents (after 1 h at rest): (**a**) hexane, (**b**) water, (**c**) 2-propanol, (**d**) ethyl acetate, and (**e**) toluene. Samples (**b**–**e**) were considered to be "GOOD"; sample (**a**) was considered to be "BAD".

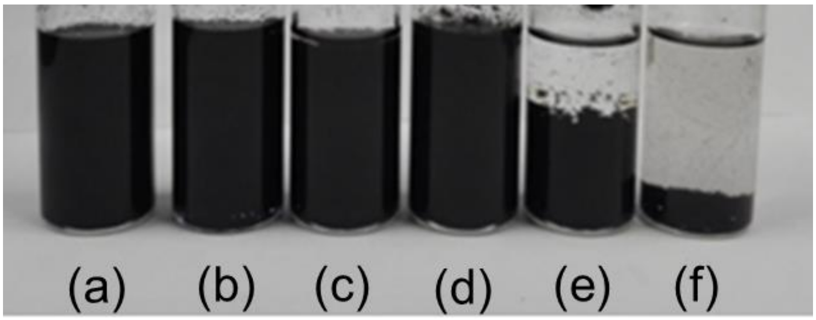

Figure S10. Dispersions of CNT-APTESP adduct in different solvents (after 1 h at rest): (**a**) 2-propanol, (**b**) dichloromethane, (**c**) ethyl acetate, (**d)** toluene, (**e**) water, and (**f**) hexane. Samples (**a**–**d**) were considered to be "GOOD"; samples (**e**,**f**) were considered to be "BAD".

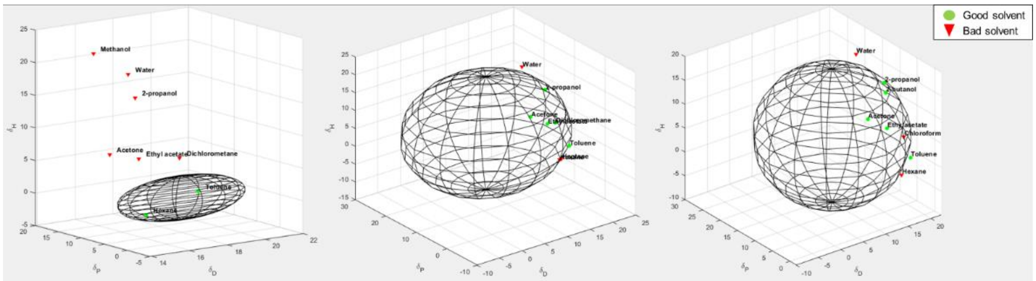

Figure S11. Hansen sphere for (**a**) CNTs; (**b**) CNT-APTESP; (**c**) CNT-TMP. The green circles correspond to the good solvents (within the radius of interaction); the red triangles correspond to the bad solvents (outside the sphere).

Table S2. Aqueous dispersions of CNT: based on CNT-SP and Carbobyk 9810.

	Carbobyk 9810	CNT-SP dispersion
**CNT type**	multiwalled	multiwalled
**CNT content**	8% w/w	4% w/w
**Solid content**	21%	4%

Carbobyk 9810 is a commercial water dispersion of CNTs produced by BYK-Chemie GmbH (Wesel, Germany). Data reported in Table S2 are taken from the technical data sheet. Further characterization by TEM and WAXD techniques has been performed. As it can be seen in the following TEM and HRTEM micrographs (Figure S12), CNTs have length from one hundred nanometers to few microns, average external diameter of about 17 nm and average internal diameter of about 5 nm. The number of walls falls between 7 and 21.

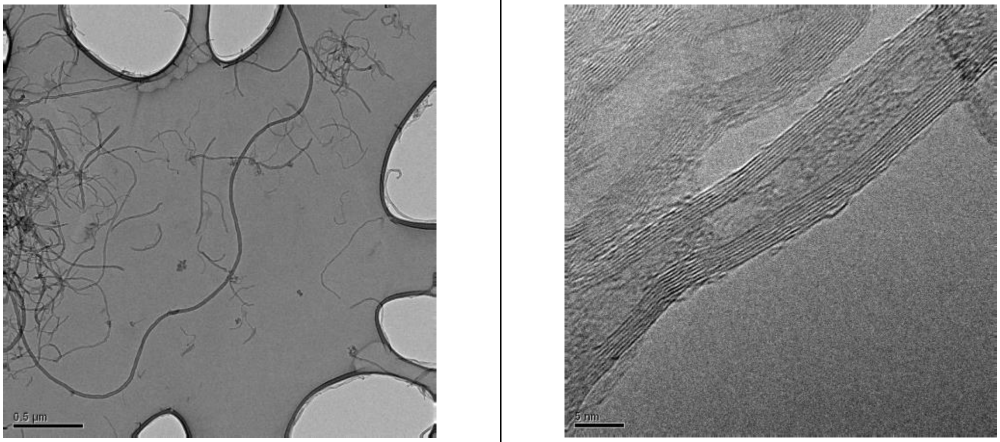

(a) (b)

Figure S12. TEM (**a**) and HRTEM (**b**) micrographs of Carbobyk 9810 MWCNT water-based dispersion.

The WAXD diffractogram reported in Figure S13 shows the (002) reflection peak typical of CNTs at about $2\theta = 25°$ that corresponds to an out-of-plane correlation length of 0.35 nm, as calculated from the Bragg's law.

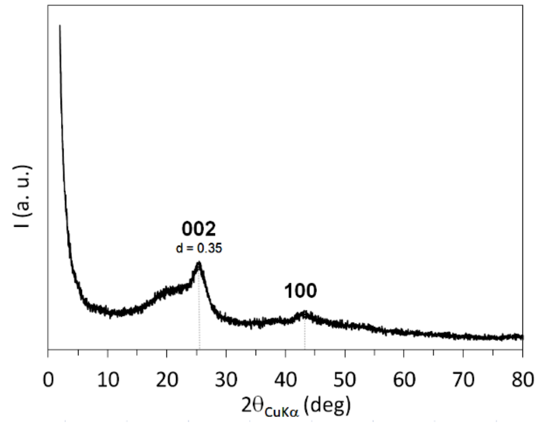

Figure S13. WAXD pattern of Carbobyk 9810 dried dispersion.

<table>
<tr><td colspan="2">Coating layers from dispersions based on</td></tr>
<tr><td>CNT-SP 4% w/w</td><td>Carbobyk9810 4% w/w</td></tr>
<tr><td>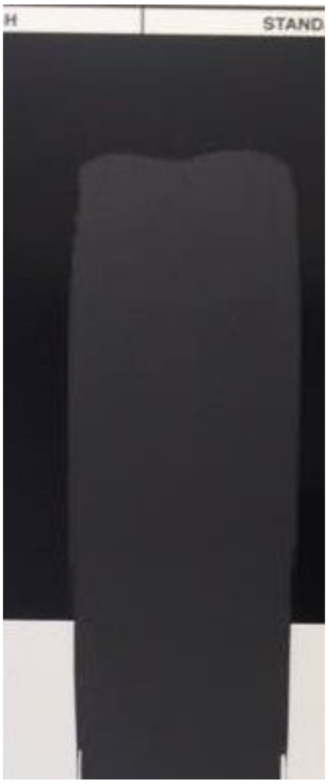
</td><td>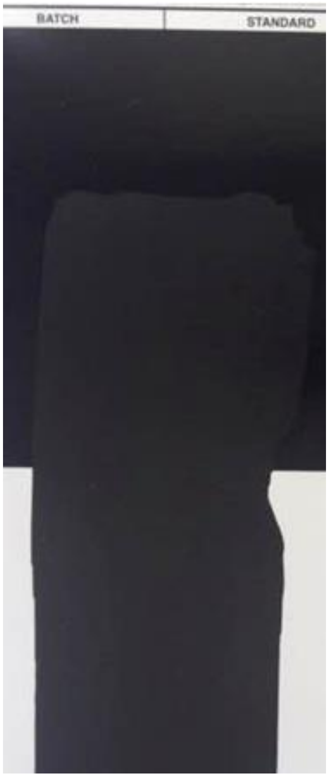
</td></tr>
</table>

Figure S14. Coating layers from dispersions based on CNT-SP adduct (left) and on Carbobyk 9810 (right). Wet coating thickness: 100 μm corresponding to 20 μm of dried coating.

References

S1 Hildebrand, J.H.; Bowers, W.G. A Study of the Action of Alkali on Certain Zinc Salts by Means of The Hydrogen Electrode. *J. Am. Chem. Soc.* **1916**, *38*, 785–788, doi:10.1021/ja02261a003.

S2 Scatchard, G. Equilibria in Non-Electrolyte Solutions in Relation to the Vapor Pressures and Densities of the Components. *Chem. Rev.* **1931**, *8*, 321–333, doi:10.1021/cr60030a010.
